# Supplementary material for: GBZ-base and GAF-base: Indexed pangenome file formats
Source: bioRxiv. 2026 Jul 11:2026.07.10.737775. Preprint. [Version 1] doi: 10.64898/2026.07.10.737775 (PMC13370461; doi:10.64898/2026.07.10.737775)
Supplement: Supplement 1 [file media-1.pdf]

# Supplement to: *GBZ-base and GAF-base: Indexed pangenome file formats*

Jouni Sirén, Benedict Paten

July 10, 2026

## 1 Data sources

### 1.1 Graphs

We used HPRC release 2 version 2.1 evaluation graphs with CHM13 as the primary reference from https://s3-us-west-2.amazonaws.com/human-pangenomics/index.html?prefix=pangenomes/scratch/2025_12_23_minigraph_cactus/benchmark-graphs/hprc-v2.1-mc-chm13-eval/. These graphs do not contain samples HG002, HG005, and NA19240.

- Default graph: hprc-v2.1-mc-chm13-eval.gbz (`default.gbz` in the scripts)
- Haplotype index for the default graph: hprc-v2.1-mc-chm13-eval.hapl (`default.hapl`)
- Frequency-filtered graph: hprc-v2.1-mc-chm13-eval.d46.gbz (`filtered.gbz`)

### 1.2 Reads

We reused the high-coverage reads for HG002 from the long read Giraffe paper [2]. The reads are available at: https://cgl.gi.ucsc.edu/data/lr-giraffe/reads/real/HG002/.

- Element Biosciences: HG002.GAT-LI-C044.fastq.gz (`element.fq.gz`). Originally from the Telomere-to-Telomere Consortium [3].
- Illumina NovaSeq: HG002.novaseq.pcr-free.40x.fq.gz (`illumina.fq.gz`). Originally from Google's gold-standard benchmarking dataset collection [1].
- PacBio HiFi: HG002Revio_hg002v1.0.1_hifi_revio_pbmay24.pri.unshuffled.fastq.gz (`hifi.fq.gz`). Originally from the Telomere-to-Telomere Consortium [3].
- ONT R10: r10y2025.HG002_PAW70337.fastq.gz (`ont.fq.gz`). Originally from the Genome in a Bottle data release 2025.01 by Oxford Nanopore Technologies [4].

## 2 The Graph Alignment Format (GAF)

The latest version of this document is hosted at:
https://github.com/vgteam/vg/blob/master/doc/static/GAF.md

This document describes version 1.0 of the vg interpretation of the Graph Alignment Format (GAF). It is a superset of a subset of the original GAF format. That format in turn is a superset of the PAF format. Sequence names and optional fields follow conventions set in the SAM format. Difference strings are defined in the minimap2 man page. Paths are represented as GFA walks. Reference graphs may have pggname stable names.

### 2.1 Overview

GAF is a tab-delimited file format for sequence alignments to bidirected sequence graphs. The file is encoded in UTF-8. Unless otherwise specified, all fields are restricted to 7-bit US-ASCII.

Each file consists of a number of header lines followed by a number of alignment lines. Each line can be split into a number of fields separated by TAB (`\t`) characters.

### 2.2 Typed fields

Typed fields are stored in the SAM-style `TAG:TYPE:VALUE` format. The tag is a two-character string matching `[A-Za-z][A-Za-z0-9]`.

The following types are currently supported:

| Type | Description |
|---|---|
| A | Printable character in `[!-~]` |
| Z | String of printable characters and spaces (`[ !-~]*`) |
| i | Signed 64-bit integer |
| f | Double-precision floating point number |
| b | Boolean value, with 1 for true and 0 for false |

### 2.3 Header lines

#### Since vg 1.70.0

Header lines are optional, and they must all appear before the first alignment line. The first field of each header line is a three-character tag matching `@[A-Za-z][A-Za-z0-9]`.

**Example:**

```
@HD VN:Z:1.0
@RN 7f4b28c71ceb808aebd8b8e9fe85e79d0d208ee263ffe9fcdef5ade20534ceb5
@SG 7f4b28c71ceb808aebd8b8e9fe85e79d0d208ee263ffe9fcdef5ade20534ceb5
    e10f3b362d8a4273059d9aea38a78bd71913418c3f3c9a2b5ea44e86de2c1181
@TL e10f3b362d8a4273059d9aea38a78bd71913418c3f3c9a2b5ea44e86de2c1181
    1f133f116e8dd98fc07a647a8954038c2bcf07a45759ba94718471fe34ed7a7c
```

#### 2.3.1 File headers

File headers start with tag `@HD`. They may contain any number of optional typed fields. The following optional fields are known.

| Tag | Type | Description |
|---|---|---|
| VN | Z | Version number (e.g. 1.0; only one allowed in the file) |

#### 2.3.2 Reference name

**Since vg 1.71.0**

The graph the sequences were aligned to can be identified using a reference name line. A reference name line starts with tag `@RN` and contains the pggname (SHA-256 hash of the canonical GFA representation) of the graph as the second field. There may be optional typed fields. There can be only one reference name line in a file.

#### 2.3.3 Graph relationships

**Since vg 1.71.0**

If the sequences were aligned to graph A, which is a subgraph of B, graph B is also a valid reference for the alignments. If there is a known coordinate translation from graph B to graph C, graph C can also be used as a reference after translating the coordinates. Subgraph (`@SG`) and translation (`@TL`) lines can used to describe such relationships between reference graphs.

A subgraph line contains the pggname of the subgraph as the second field and the name of the supergraph as the third field. A translation line contains the name of the source graph as the second field and the name of the destination graph as the third field. Both line types may contain optional typed fields, and there may be any number of such lines.

### 2.4 Alignment lines

Each alignment line has 12 mandatory fields. Missing values in fields 3 to 11 are indicated by character `*`.

| Field | Type | Description |
|---|---|---|
| 1 | Z | Query sequence name |
| 2 | i | Query sequence length |
| 3 | i | Query start (0-based; closed) |
| 4 | i | Query end (0-based; open) |
| 5 | A | Strand relative to the path; always + |
| 6 | Z | Target path represented as a GFA walk |
| 7 | i | Target path length |
| 8 | i | Start position on the target path (0-based; closed) |
| 9 | i | End position on the target path (0-based; open) |
| 10 | i | Number of matches |
| 11 | i | Number of matches, mismatches, insertions, and deletions |
| 12 | i | Mapping quality (0-255; 255 for missing) |

**Example:**

```
read1   6   0   6   +   >2>3>4  12  2   8   6   6   60  cs:Z::6
read2   7   0   7   +   >2>5>6  11  1   8   7   7   60  cs:Z::7
read3   7   0   7   *   *   *   *   *   *   *   255 cs:Z:+GATTACA
```

### 2.4.1 Query sequence name

Query sequence names must follow SAM conventions. A name may contain any printable ASCII characters in the range `[!-~]`, except `@`. This allows distinguishing header lines from alignment lines.

### 2.4.2 Target path

This version of GAF does not allow specifying the target path using stable rGFA coordinates or nodes (GFA segments) with string names. Nodes must have positive integer identifiers. Node identifier `0` cannot be used, as many graph implementations reserve it for technical purposes.

### 2.4.3 Optional fields

Optional fields are SAM-style typed fields. No tag can appear more than once on the same line, and the order of the optional fields does not matter.

### 2.4.4 Difference string

Difference strings represent an edit script that transforms the given interval of the target path to the given interval of the query sequence. They are stored as an optional field `cs` of type `Z`. We support a subset of the operations defined for minimap2 difference strings.

| Operation | Regex | Description |
|---|---|---|
| : | [0-9]+ | Number of matching bases |
| * | [ACGTN][ACGTN] | Mismatch as (target base, query base) |
| + | [ACGTN]+ | Insertion as the unaligned query bases |
| - | [ACGTN]+ | Deletion as the unaligned target bases |

### 2.4.5 Other defined optional fields

| Tag | Type | Description |
|---|---|---|
| AS | i | Alignment score |
| bq | Z | Base quality string; must have the same length as the query sequence |
| fn | Z | Name of the next fragment (for paired alignments; cannot be used with `fp`) |
| fp | Z | Name of the previous fragment (for paired alignments; cannot be used with `fn`) |
| pd | b | This alignment and its pair (specified by `fn` or `fp`) are properly paired |
| fi | i | Fragment identifier for a fragmented alignment (see below) |

## 2.5 Conventions

### 2.5.1 Header lines

**Since vg 1.70.0**

The first line of a GAF file is a file header (`@HD`) with the version number (`VZ:Z`) tag. Any additional file header lines follow. Other types of header lines are after file header lines.

### 2.5.2 Primary alignments

A primary alignment represents an alignment of the entire query sequence to a non-empty interval of a target path. Query start (field 3) must be `0` and query end (field 4) must have the same value as query sequence length (field 2). A difference string must be present to allow recovering the entire query sequence.

### 2.5.3 Unaligned sequences

**Since vg 1.70.0**

An unaligned sequence is represented as an alignment of the entire query sequence to a missing interval of a missing target path. Query start (field 3) must be `0` and query end (field 4) must have the same value as query sequence length (field 2). A difference string must be present, with the entire query sequence as a single insertion, to allow recovering the sequence.

### 2.5.4 Fragmented alignments

A fragmented alignment is a single alignment represented as number of alignment lines (e.g. corresponding to subpaths that are within a specific subgraph). The fragments (alignment lines) correspond to non-overlapping intervals of the underlying alignment. Each fragment represents an alignment of a non-empty query interval to a non-empty interval of a non-empty target path.

Query interval (fields 3 and 4), target path (fields 6 to 9), and the difference string must be specific to each fragment. Alignment statistics (fields 10 to 12) may be inherited from the underlying alignment or be specific to each fragment. Fragments are identified by fragment indexes starting from `1`, stored as an optional field `fi` of type `i`.

# 3 Stable names for pangenome graphs

The latest version of this document is hosted at:
https://github.com/jltsiren/pggname/blob/main/README.md

This is a proposal for generating stable names for pangenome graphs. The names are SHA-256 hashes of a canonical GFA representation of the graph.

See refget for a similar naming scheme for sequences.

### 3.1 Intended applications

- Tagging various indexes with the name of the corresponding graph.
- As a reference name in a read alignment file.
- For representing relationships such as "A is a subgraph of B" or "A can be translated to B".
  - If A is a subgraph of B, graph B can be used as a reference with reads aligned to A.
  - Some tools chop long nodes to smaller fragments, but coordinates in the chopped graph can be translated to the original coordinates.

### 3.2 Example

We have three graphs:

- `original.gfa`: The original graph with some long nodes.
- `translated.gbz`: The same graph, with long nodes chopped into 1024 bp fragments.
- `sampled.gbz`: A personalized graph sampled from `translated.gbz`.

These graphs have the following names:

```
1f133f116e8dd98fc07a647a8954038c2bcf07a45759ba94718471fe34ed7a7c
    original.gfa
e10f3b362d8a4273059d9aea38a78bd71913418c3f3c9a2b5ea44e86de2c1181
    translated.gbz
7f4b28c71ceb808aebd8b8e9fe85e79d0d208ee263ffe9fcdef5ade20534ceb5
    sampled.gbz
```

We want to store the following information for `sampled.gbz`:

- The name of the graph.
- `sampled.gbz` is a subgraph of `translated.gbz`.
- Coordinates can be translated in both directions between `translated.gbz` and `original.gfa`.

#### 3.2.1 GBZ tags

```
pggname = 7f4b28c71ceb808aebd8b8e9fe85e79d0d208ee263ffe9fcdef5ade20534ceb5
subgraph = 7f4b28c71ceb808aebd8b8e9fe85e79d0d208ee263ffe9fcdef5ade20534ceb5,
    e10f3b362d8a4273059d9aea38a78bd71913418c3f3c9a2b5ea44e86de2c1181
translation = e10f3b362d8a4273059d9aea38a78bd71913418c3f3c9a2b5ea44e86de2c1181,
    1f133f116e8dd98fc07a647a8954038c2bcf07a45759ba94718471fe34ed7a7c;
    1f133f116e8dd98fc07a647a8954038c2bcf07a45759ba94718471fe34ed7a7c,
    e10f3b362d8a4273059d9aea38a78bd71913418c3f3c9a2b5ea44e86de2c1181
```

#### 3.2.2 GFA header

```
H   NM:Z:7f4b28c71ceb808aebd8b8e9fe85e79d0d208ee263ffe9fcdef5ade20534ceb5
H   SG:Z:7f4b28c71ceb808aebd8b8e9fe85e79d0d208ee263ffe9fcdef5ade20534ceb5,
    e10f3b362d8a4273059d9aea38a78bd71913418c3f3c9a2b5ea44e86de2c1181
H   TL:Z:e10f3b362d8a4273059d9aea38a78bd71913418c3f3c9a2b5ea44e86de2c1181,
    1f133f116e8dd98fc07a647a8954038c2bcf07a45759ba94718471fe34ed7a7c
H   TL:Z:1f133f116e8dd98fc07a647a8954038c2bcf07a45759ba94718471fe34ed7a7c,
    e10f3b362d8a4273059d9aea38a78bd71913418c3f3c9a2b5ea44e86de2c1181
```

#### 3.2.3 GAF header

```
@RN 7f4b28c71ceb808aebd8b8e9fe85e79d0d208ee263ffe9fcdef5ade20534ceb5
@SG 7f4b28c71ceb808aebd8b8e9fe85e79d0d208ee263ffe9fcdef5ade20534ceb5
    e10f3b362d8a4273059d9aea38a78bd71913418c3f3c9a2b5ea44e86de2c1181
@TL e10f3b362d8a4273059d9aea38a78bd71913418c3f3c9a2b5ea44e86de2c1181
    1f133f116e8dd98fc07a647a8954038c2bcf07a45759ba94718471fe34ed7a7c
@TL 1f133f116e8dd98fc07a647a8954038c2bcf07a45759ba94718471fe34ed7a7c
    e10f3b362d8a4273059d9aea38a78bd71913418c3f3c9a2b5ea44e86de2c1181
```

Here we use `RN` (reference name) instead of `NM` (name).

### 3.3 Canonical GFA format

Sort the nodes by their identifiers. Interpret node identifiers as integers, if possible, and fall back to strings if at least one of the identifiers is not an integer.

For each node, in sorted order, output:

- S-line for the node without optional fields.
- L-lines for all canonical edges, without the overlap field or optional fields, in sorted order.

The canonical GFA representation of the graph does not include any other information, such as header lines, paths, or walks.

An edge is canonical, if the source id is smaller than the destination id. A self-loop is canonical, if at least one of the nodes is in forward orientation.

Edges are sorted by (source orientation, destination id, destination orientation). The forward orientation comes before the reverse orientation.

#### 3.3.1 Example

Consider the following example graph from the GFA specification, with overlaps changed to `0M`:

```
H   VN:Z:1.0
S   11  ACCTT
```

| Dataset | GBWT | Start | Names | Quality | Difference | Flags | Numbers | Optional |
|---|---|---|---|---|---|---|---|---|
| Element | 3.998 | 2.970 | 9.599 | 60.570 | 2.691 | 1.135 | 7.531 | 0.570 |
| Illumina | 3.965 | 2.184 | 5.737 | 7.372 | 2.484 | 0.773 | 3.688 | 0.442 |
| HiFi | 3.600 | 0.031 | 0.140 | 15.962 | 1.531 | 0.011 | 0.062 | 0.064 |
| ONT | 3.647 | 0.020 | 0.182 | 42.958 | 5.716 | 0.007 | 0.036 | 0.049 |

Table 1: GAF-base size breakdown, with the size of each component in gibibytes ($2^{30}$ bytes). Target paths stored in table `Nodes`, GBWT positions for path starts, read/pair names, quality strings, difference strings, binary flags, numbers, and optional fields.

```
S   12  TCAAGG
S   13  CTTGATT
L   11  +   12  -   0M
L   12  -   13  +   0M
L   11  +   13  +   0M
P   14  11+,12-,13+ 0M,0M
```

Its canonical GFA representation is:

```
S   11  ACCTT
L   11  +   12  -
L   11  +   13  +
S   12  TCAAGG
L   12  -   13  +
S   13  CTTGATT
```

And its stable name is:

```
54b49d18354a34fbd1af9aaac279e1b3ee67b2f68f0ff79f5ebf6c50c8d922a5
```

## 4 GAF-base size breakdown

The major components of a GAF-base are the `Nodes` table storing the target paths and the binary blobs in table `Alignments`. Table 1 lists the size breakdown between those components for each dataset. For the target paths, we measure the size of the database file before and after inserting the data into the `Nodes` table. For the other components, we measure the total size of the binary blobs stored in the corresponding fields, excluding database overhead.

Quality strings are the largest single component for all datasets. For the other datasets except Illumina, they require more space than all the other components combined. The space used for difference strings depends on the error profile of the sequencing technology, being smallest with HiFi and largest with ONT. Target paths use similar space for all datasets, as the size of a GBWT index depends mostly on the number of node records. GBWT starting positions require 2 bytes/alignment in most cases. Read / pair names and numerical fields use substantial space with short reads and negligible space with long reads.

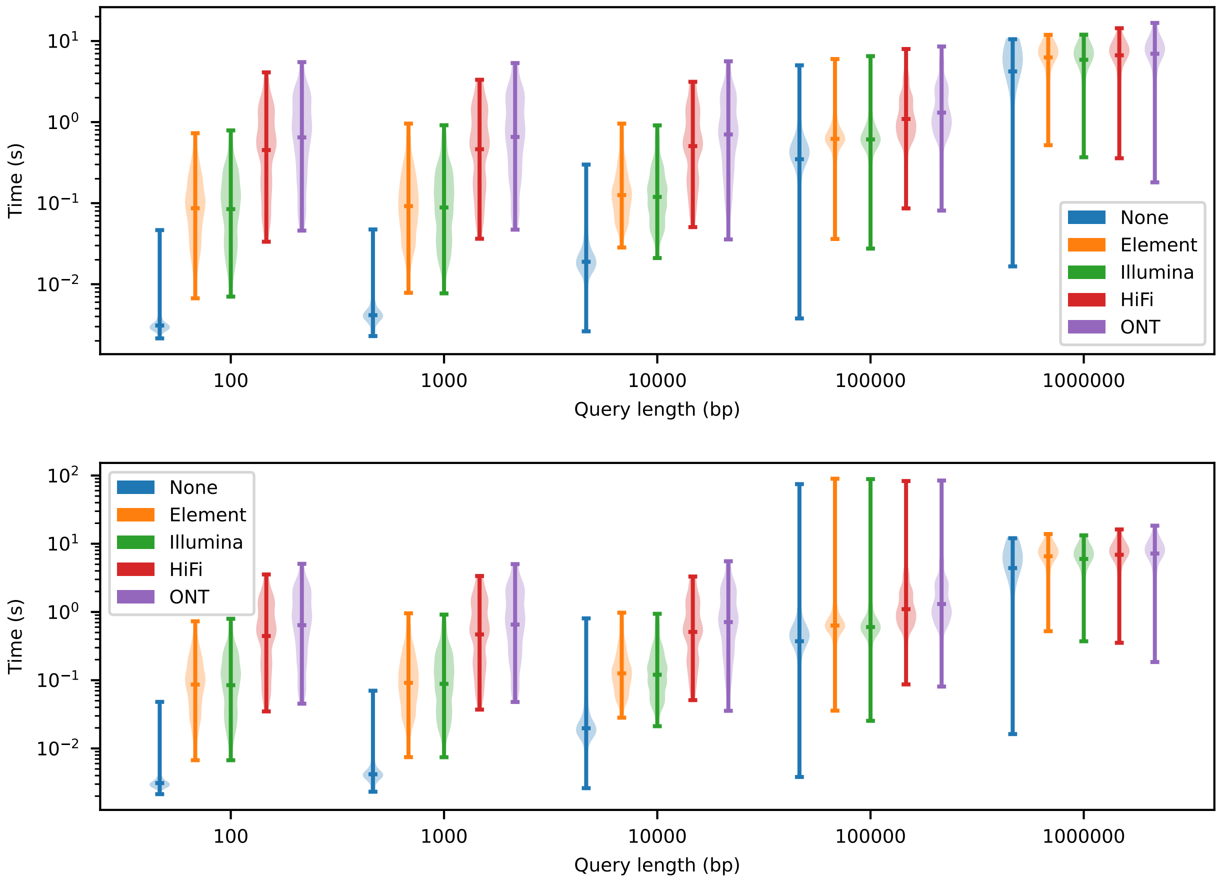


Figure 1: Violin plots for subgraph query times with random CHM13 intervals and 100 bp greedy context. Queries without snarls (top) and with contained snarls (bottom). GBZ-base with no reads and with various GAF-bases.

## 5 Subgraph queries

Full subgraph query results can be found in Figure 1. When we do not extend the subgraph with snarls, we avoid the outliers caused by large deletions within the greedy context. Apart from those outliers, query times are similar with and without snarls. Query performance is similar with Element and Illumina reads. Queries using ONT reads are somewhat slower than those with HiFi reads.

Peak memory usage for each query session can be found in Table 2. The memory usage of an individual query depends on the effective size of the subgraph (including the handles used by candidate alignments) and on the number and the length of the alignments in the subgraph. Peak memory usage is the maximum over the queries in the session.

Figure 2 shows the number of handles in the subgraph and in the candidate alignments with Illumina and HiFi reads. The latter is the effective size of the subgraph we must extract from GBZ-base when we want to extract the alignments overlapping with the subgraph. With 100 bp queries, this effective subgraph is orders of magnitude larger than the actual subgraph. This explains why there is little difference in query times between 100 bp and 10 kbp queries when we also extract alignments from a GAF-base.

| Reads | Snarls | 100 bp | 1000 bp | 10000 bp | 100000 bp | 1000000 bp |
|---|---|---|---|---|---|---|
| None | None | 26.047 MiB | 37.484 MiB | 170.828 MiB | 1.804 GiB | 2.281 GiB |
| None | Contained | 25.891 MiB | 41.891 MiB | 316.453 MiB | 9.187 GiB | 2.293 GiB |
| Element | None | 56.781 MiB | 65.062 MiB | 204.484 MiB | 2.339 GiB | 3.253 GiB |
| Element | Contained | 56.750 MiB | 72.016 MiB | 365.031 MiB | 12.392 GiB | 3.072 GiB |
| Illumina | None | 59.250 MiB | 65.594 MiB | 195.938 MiB | 2.134 GiB | 2.838 GiB |
| Illumina | Contained | 59.625 MiB | 72.859 MiB | 352.219 MiB | 10.928 GiB | 2.906 GiB |
| HiFi | None | 180.000 MiB | 176.188 MiB | 301.766 MiB | 2.136 GiB | 2.649 GiB |
| HiFi | Contained | 179.875 MiB | 180.219 MiB | 440.344 MiB | 9.319 GiB | 2.702 GiB |
| ONT | None | 229.484 MiB | 239.922 MiB | 374.078 MiB | 1.996 GiB | 2.510 GiB |
| ONT | Contained | 228.469 MiB | 247.828 MiB | 507.375 MiB | 9.215 GiB | 2.552 GiB |

Table 2: Peak memory usage (peak resident set size) for each query session.

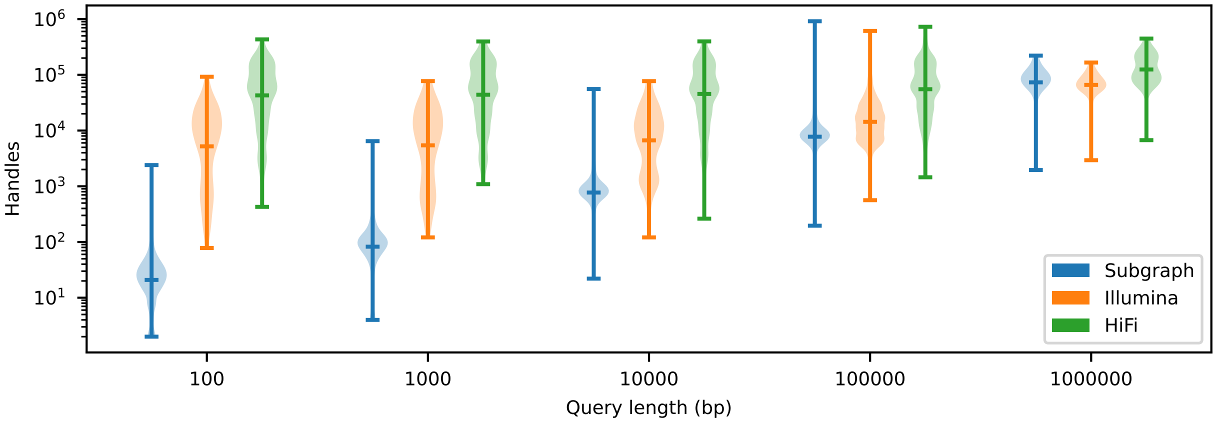


Figure 2: Violin plots for the number of handles in the subgraph and in the candidate alignments of Illumina / HiFi reads with random CHM13 intervals, 100 bp greedy context, and contained snarls.

## 6 Specific commands used

We assume that the following directories are located on a fast local SSD:

- `$WORK`: Working directory containing the graphs and the reads
- `$TMPDIR`: Temporary directory
- `./logs`: Logs

All relevant tools are assumed to be found in the path. GBZ-base / GAF-base tools are compiled with:

```
cargo build --release --features=benchmark
```

Variable `$1` in the scripts is the name of the dataset (e.g. `illumina`).

## 6.1 GBZ-base construction

We copied the default graph `default.gbz` to the current directory. Then we build GBZ-base with:

```
LOGFILE=logs/gbz-base-${1}.log
rm -f $LOGFILE

gbz2db --overwrite ${1}.gbz 2>> $LOGFILE
```

The log contains wall-clock time and peak memory usage for the construction, as well as the size of the GBZ-base database.

## 6.2 GAF-base construction

### 6.2.1 Mapping reads with Giraffe

We use the following script for mapping the reads:

```
if [ "$1" == element ]; then
  GRAPH=${WORK}/default.gbz
  KMERS=${WORK}/${1}.kff
  SAMPLING="--haplotype-name ${WORK}/default.hapl --kff-name $KMERS"
  PAIRED="-i"
elif [ "$1" == illumina ]; then
  GRAPH=${WORK}/default.gbz
  KMERS=${WORK}/${1}.kff
  SAMPLING="--haplotype-name ${WORK}/default.hapl --kff-name $KMERS"
  PAIRED="-i"
elif [ "$1" == hifi ]; then
  GRAPH=${WORK}/filtered.gbz
  PRESET="--parameter-preset hifi"
elif [ "$1" == ont ]; then
  GRAPH=${WORK}/filtered.gbz
  PRESET="--parameter-preset r10"
fi

LOGFILE=logs/giraffe-${1}.log
rm -f $LOGFILE

INPUT=${WORK}/${1}.fq.gz
OUTPUT=${WORK}/${1}.gaf.gz
THREADS=32

if [ "$KMERS" != "" ]; then
  kmc -k29 -m128 -okff -t${THREADS} -hp $INPUT ${WORK}/${1} $TMPDIR
fi

vg giraffe -p -t $THREADS -Z $GRAPH $SAMPLING $PAIRED -f $INPUT $PRESET -o gaf \
  2>> $LOGFILE | bgzip > $OUTPUT
```

With short reads (Element and Illumina), we do paired-end mapping to a personalized graph. We count $k$-mers separately with `kmc` and let `vg giraffe` run haplotype sampling and build indexes for the personalized graph. While `vg giraffe` could also run `kmc` automatically, there is a bug in vg version 1.75.0 that makes the alignment output incorrect when option `-p` / `--progress` is used. This bug has been fixed in vg version 1.75.1.

With long reads (HiFi and ONT), we use the appropriate preset and map the reads to the frequency-filtered graph. `vg giraffe` builds the indexes automatically if they do not exist. This assumes that the datasets are mapped one at a time. If both datasets are mapped concurrently, the indexes should be built first with `vg autoindex` to avoid conflicts between multiple jobs building the same indexes.

#### 6.2.2 GAF sorting

We use the following script for sorting the original GAF file:

```
if [ "$1" == element ]; then
  SORT_BLOCK=1M
  DB_BLOCK=1000
elif [ "$1" == illumina ]; then
  SORT_BLOCK=1M
  DB_BLOCK=1000
elif [ "$1" == hifi ]; then
  SORT_BLOCK=10k
  DB_BLOCK=10
elif [ "$1" == ont ]; then
  SORT_BLOCK=10k
  DB_BLOCK=10
fi

LOGFILE=logs/gaf-base-${1}.log
rm -f $LOGFILE

INPUT=${WORK}/${1}.gaf.gz
OUTPUT=${WORK}/${1}.sorted.gaf.gz
DB=${WORK}/${1}.db
GRAPH=${WORK}/default.gbz
DECOMPRESSED=${WORK}/temp.gaf.gz
THREADS=16
BGZIP_THREADS=6

gafsort -p -r $SORT_BLOCK -t $THREADS $INPUT 2>> $LOGFILE \
  | bgzip --threads $BGZIP_THREADS > $OUTPUT
```

The number of sorting threads primarily affects the intermediate merging rounds. The final merge is mostly sequential, while a few threads are already enough to saturate the gzip decompression in `gafsort` during the initial sorting. Wall-clock time and peak memory usage can be found in the log.

#### 6.2.3 GAF-base construction

The script continues with GAF-base construction:

```
echo >> $LOGFILE

gaf2db --overwrite -b $DB_BLOCK -o $DB $OUTPUT 2>> $LOGFILE
```

Wall-clock time and peak memory usage can be found in the log again. The construction also reports the size of the database after creating each table, as well as the total sizes of various fields in table `Alignments`. GAF-base size breakdown (Section 4) can be derived from this information.

#### 6.2.4 GAF-base decompression

The final part of the script is GAF-base decompression:

```
echo >> $LOGFILE

(db2gaf -r $GRAPH $DB 2>> $LOGFILE) \
  | bgzip --threads $BGZIP_THREADS > $DECOMPRESSED
rm -f $DECOMPRESSED
```

Wall-clock time and peak memory usage can be found in the log. We need a reference graph for converting the internal representation of the difference strings to actual difference strings that in some cases contain the reference bases corresponding to the edit operations.

### 6.3 GAF-base size comparison

We determine the file sizes for each dataset using the following script:

```
if [ "$1" == element ]; then
  READ_LENGTH="--read-length short"
elif [ "$1" == illumina ]; then
  READ_LENGTH="--read-length short"
elif [ "$1" == hifi ]; then
  READ_LENGTH="--read-length long"
elif [ "$1" == ont ]; then
  READ_LENGTH="--read-length long"
fi

LOGFILE=logs/file-sizes-${1}.log
rm -f $LOGFILE

BASENAME=${WORK}/${1}
GRAPH=${WORK}/default.gbz
REFERENCE=${WORK}/chm13.fa
REFERENCE_SAMPLE=CHM13
THREADS=32
```

```
# Extract the correct reference, if necessary.
if [ ! -f $REFERENCE ]; then
  vg paths -x $GRAPH -S $REFERENCE_SAMPLE -F > $REFERENCE
  samtools faidx $REFERENCE
fi

# Compressed GAF
ls -l ${BASENAME}.gaf.gz >> $LOGFILE

# Sorted compressed GAF
ls -l ${BASENAME}.sorted.gaf.gz >> $LOGFILE

# GAF-base
ls -l ${BASENAME}.db >> $LOGFILE

# GAF
bgzip -d -c --threads $THREADS ${BASENAME}.gaf.gz > ${BASENAME}.gaf
ls -l ${BASENAME}.gaf >> $LOGFILE
rm -f ${BASENAME}.gaf

# GAM
vg convert -t $THREADS --gaf-to-gam ${BASENAME}.gaf.gz $GRAPH > ${BASENAME}.gam
ls -l ${BASENAME}.gam >> $LOGFILE
rm -f ${BASENAME}.gam

# BAM
vg surject -t $THREADS -x $GRAPH --into-ref $REFERENCE_SAMPLE $READ_LENGTH \
  --gaf-input --bam-output ${BASENAME}.gaf.gz > ${BASENAME}.bam
ls -l ${BASENAME}.bam >> $LOGFILE

# Sorted CRAM (from BAM)
samtools sort --threads $THREADS -m 4G -T ${TMPDIR}/samtools ${BASENAME}.bam \
  > ${BASENAME}.sorted.bam
samtools view --threads $THREADS --reference $REFERENCE \
  --cram ${BASENAME}.sorted.bam > ${BASENAME}.cram
ls -l ${BASENAME}.cram >> $LOGFILE
rm -f ${BASENAME}.bam ${BASENAME}.sorted.bam ${BASENAME}.cram
```

We convert GAF to GAM with `vg convert` and GAF to BAM with `vg surject`. Then we obtain sorted CRAM from BAM by sorting it with `samtools sort` and converting the result with `samtools view`.

Since we used SAMtools 1.19.2 with default parameters, the CRAM files were of version 3.0. CRAM 3.1 files would have been smaller due to the availability of better codecs.

### 6.3.1 Subgraph queries

For benchmarking subgraph queries, we copied all GAF-bases to the current directory. Before each session, we purged disk caches with `sudo purge`. We ran `query-benchmark` with the following options:

- `--gbz-base default.gbz.db`
- With no GAF-base and with `--gaf-base {$1}.db` for each dataset `$1`
- Using CHM13 as the reference: `--faidx CHM13.fa.fai --sample CHM13`
- `--interval-length $N` for `$N` in $\{100, 1000, 10000, 100000, 1000000\}$
- `--num-queries $M`, with `$M` as 1000 otherwise and as 100 when `$N` was 1000000
- With and without `--snarls`
- `--verbose`

We redirected the standard output to a log file. For each query, the log contains a line with the following TAB-separated fields:

1. Description of the query.
2. Number of nodes in the subgraph.
3. Number of alignment fragments in the output.
4. Number of original alignments in the subgraph.
5. Number of alignment blocks decompressed.
6. Number of candidate alignments in the blocks.
7. Number of distinct handles in the candidates.
8. Query time in seconds.

## References


[1] G. Baid et al. An extensive sequence dataset of gold-standard samples for benchmarking and development. bioRxiv, 2020.

[2] X. Chang et al. Rapid, accurate long- and short-read mapping to large pangenome graphs with vg Giraffe. bioRxiv, 2025.

[3] N. F. Hansen et al. A complete diploid human genome benchmark for personalized genomics. bioRxiv, 2025.

[4] Oxford Nanopore Technologies. Genome in a Bottle data release 2025.01. https://epi2me.nanoporetech.com/giab-2025.01/, 2025.
